# Selective JAK2/ABL dual inhibition therapy effectively eliminates TKI-insensitive CML stem/progenitor cells

**DOI:** 10.18632/oncotarget.2353

**Published:** 2014-08-16

**Authors:** Hanyang Lin, Min Chen, Katharina Rothe, Matthew V. Lorenzi, Adrian Woolfson, Xiaoyan Jiang

**Affiliations:** ^1^ Terry Fox Laboratory, British Columbia Cancer Agency, University of British Columbia; Vancouver, BC, Canada V5Z 1L3; ^2^ Department of Medicine, University of British Columbia, University of British Columbia; Vancouver, BC, Canada V5Z 1L3; ^3^ Department of Medical Genetics, University of British Columbia; Vancouver, BC, Canada V5Z 1L3; ^4^ Discovery Medicine Oncology, Bristol-Myers Squibb, Princeton, NJ, United States

**Keywords:** CML, BCR-ABL, leukemic stem cells, JAK2, TKI resistance, BMS-911543

## Abstract

Imatinib Mesylate (IM) and other tyrosine kinase inhibitor (TKI) therapies have had a major impact on the treatment of chronic myeloid leukemia (CML). However, TKI monotherapy is not curative, with relapse and persistence of leukemic stem cells (LSCs) remaining a challenge. We have recently identified an AHI-1-BCR-ABL-JAK2 protein complex that contributes to the transforming activity of BCR-ABL and IM-resistance in CML stem/progenitor cells. JAK2 thus emerges as an attractive target for improved therapies, but off-target effects of newly developed JAK2 inhibitors on normal hematopoietic cells remain a concern. We have examined the biological effects of a highly selective, orally bioavailable JAK2 inhibitor, BMS-911543, in combination with TKIs on CD34^+^ treatment-naïve IM-nonresponder cells. Combination therapy reduces JAK2/STAT5 and CRKL activities, induces apoptosis, inhibits proliferation and colony growth, and eliminates CML LSCs *in vitro*. Importantly, BMS-911543 selectively targets CML stem/progenitor cells while sparing healthy stem/progenitor cells. Oral BMS-911543 combined with the potent TKI dasatinib more effectively eliminates infiltrated leukemic cells in hematopoietic tissues than TKI monotherapy and enhances survival of leukemic mice. Dual targeting BCR-ABL and JAK2 activities in CML stem/progenitor cells may consequently lead to more effective disease eradication, especially in patients at high risk of TKI resistance and disease progression.

## INTRODUCTION

Chronic myeloid leukemia (CML) is a lethal hematological malignancy defined by the presence of a BCR-ABL fusion gene originating in a hematopoietic stem cell (HSC) [1, 2]. The BCR-ABL oncoprotein has constitutively active tyrosine kinase (TK) activity, which drives the disease phenotype by perturbing multiple signaling pathways, including PI3K/AKT, RAS/MAPK, and JAK2/STAT5 [3, 4]. The treatment of chronic phase CML (CP-CML) has been significantly improved by Imatinib Mesylate (IM) and other tyrosine kinase inhibitor (TKI) therapies [5-9]. TKI monotherapies are not, however, curative and early relapse and primary and acquired TKI resistance remain significant issues [10, 11]. Despite the effectiveness of TKI monotherapy, most patients harbor residual leukemic stem cells (LSCs), and disease typically recurs if therapy is discontinued [12, 13]. Furthermore, 15-20% of patients with early CP-CML and up to 40% with accelerated phase (AP-CML) disease fail treatment, indicating a need for alternative approaches. LSCs are known to be genetically unstable and less responsive to TKI treatments, and are of critical importance in mediating TKI resistance [14-17]. Recent studies have indicated that CML LSCs might not be exclusively dependent on BCR-ABL TK activity for their survival [18, 19]. These observations emphasize the need to develop new therapeutic agents and combination strategies to target TKI resistant LSC subclones.

Evidence suggests that the Janus kinase 2/signal transducers and activators of transcription 5 (JAK2/STAT5) pathway plays a critical role in CML leukemogenesis [3, 4]. In particular, JAK2 interacts directly with the C-terminal region of BCR-ABL and is a key interaction partner of BCR-ABL in CML [20, 21]. This complex enhances BCR-ABL TK activity and disrupts BCR-ABL-mediated signaling in BCR-ABL^+^ cells, possibly through direct phosphorylation of tyrosine 177 of BCR-ABL by JAK2 [20, 21]. We have recently identified an AHI-1-BCR-ABL-JAK2 protein complex that contributes both to the transforming activity of BCR-ABL and to IM-resistance in CML stem/progenitor cells. Disrupting this complex results in the elimination of IM-resistant BCR-ABL^+^ cells and primary CML stem/progenitor cells [22, 23]. Similarly, STAT5, a direct substrate of JAK2, is constitutively active in BCR-ABL-transduced cells [24, 25], and over-expression of Stat5 in murine BM cells generates a disease phenotype closely resembling BCR-ABL-induced CML [26]. High STAT5 levels were also found to mediate acquired IM resistance in CML cells and STAT5 inhibitors reduced their survival [27, 28]. Thus, targeting JAK2/STAT5 activity is consequently rational and complementary to the inhibition of BCR-ABL TK activity in CML stem/progenitor cells. Several JAK2 inhibitors are currently in various stages of clinical development in myeloproliferative neoplasms [29], including myelofibrosis [30, 31] and AML [32], but their off-target effects on healthy primitive hematopoietic cells remains challenging [29, 33, 34]. Here, we examine the potential relevance of JAK2 inhibition in CML by examining the biological effects of a highly selective, orally bioavailable JAK2 inhibitor BMS-911543 alone and in combination with second generation TKIs, including dasatinib (DA), on CD34^+^ treatment-naïve IM-nonresponder cells. We demonstrate that dual inhibition of JAK2/STAT5 and BCR-ABL is more effective in eliminating CML LSCs, but not their healthy counterparts, than TKIs alone *in vitro*, and significantly enhances progression free survival in mice.

## RESULTS

### JAK2 inhibitor in combination with IM is more effective in reducing JAK2/STAT5 activity and inhibiting proliferative capacity of IM-insensitive CML cells

**Figure 1:**
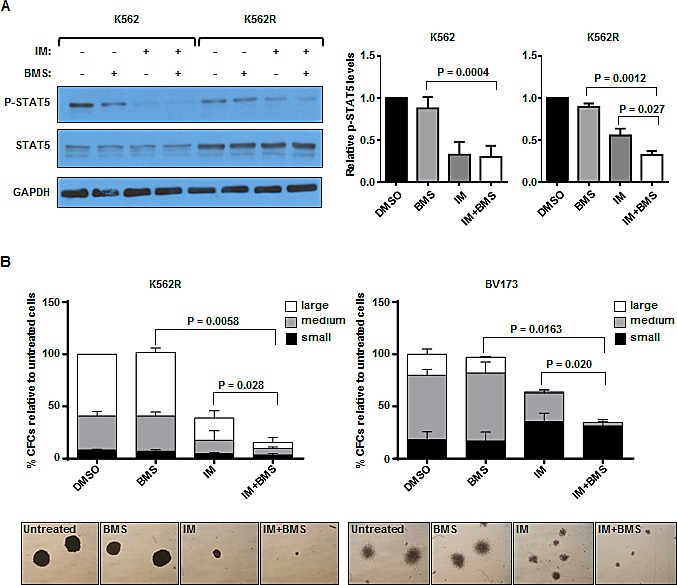
Combination treatment with BMS-911543 and imatinib (IM) is more effective at reducing pSTAT5 levels and inhibiting proliferative capacity of IM-resistant K562 and BV173 cells (A) Western blotting analysis of p-STAT5 and STAT5 in K562 and K562 IM-resistant (K562R) cells cultured with or without IM (0.05 μM), BMS-911543 (1 μM), or a combination of IM and BMS-911543 for 2 hours (left panel). DMSO was used as a control. Protein expression of p-STAT5 relative to GAPDH was compared (right panel). Data shown are mean ± SEM of measurements from three independent experiments. (B) K562R and BV173 cells were plated in standard colony-forming cell (CFC) assays plus IM (2.5 μM for K562R; 0.5 μM for BV173) or BMS-911543 (5 μM) alone or in combination. Colonies produced were counted after 16 days of incubation, and the numbers obtained were expressed as a percentage of values obtained in untreated cells to which only DMSO was added (top panel). Colony numbers for large (>500 cells), medium (50-500 cells), and small (<50 cells) are indicated. Representative photos of the size and morphology of colonies in each treatment is shown (bottom panel). Data shown are mean ± SEM of measurements from three independent experiments. *P* values were calculated using a two-tailed paired Student's *t* test.

To determine the effect of a highly selective JAK2 inhibitor (BMS-911543) [35] alone or in combination with IM on CML cells, we examined changes in the phosphorylation of STAT5, which is activated by JAK2 kinase and can be used as a measure of JAK2 kinase activity. Phosphorylated STAT5 was analyzed by Western blot analysis in K562 cells and a spontaneously-derived cell line that is relatively resistant to IM (K562R), but has no BCR-ABL kinase domain mutations [36]. Combination treatment of BMS-911543 and IM was more effective at reducing p-STAT5 levels in K562R cells compared with IM or BMS-911543 alone (70% *vs.* 45% and 10% reduction, P<0.03, Fig. 1A). This combination effect was not observed in IM-sensitive K562 cells. Increased STAT5 protein expression was observed in K562R cells as compared with IM-sensitive K562 cells. BMS-911543 and IM together also resulted in a greater reduction than IM alone in both total colony numbers and colony size produced from K562R cells in CFC assays (2-3 fold, P=0.028, Fig. 1B). Similar results were obtained from BV173 cells, a cell line derived from a CML blast crisis patient (P=0.02, Fig. 1B). These results indicate that the combination of BMS-911543 and IM result in a deeper suppression of p-STAT5 and more effectively reduce the proliferative capacity of IM-resistant cells than either single agent alone.

### The combination of BMS-911543 and TKIs reduces BCR-ABL and JAK2/STAT5 activity and induces apoptosis of CD34^+^ treatment-naïve IM-nonresponder cells

**Figure 2:**
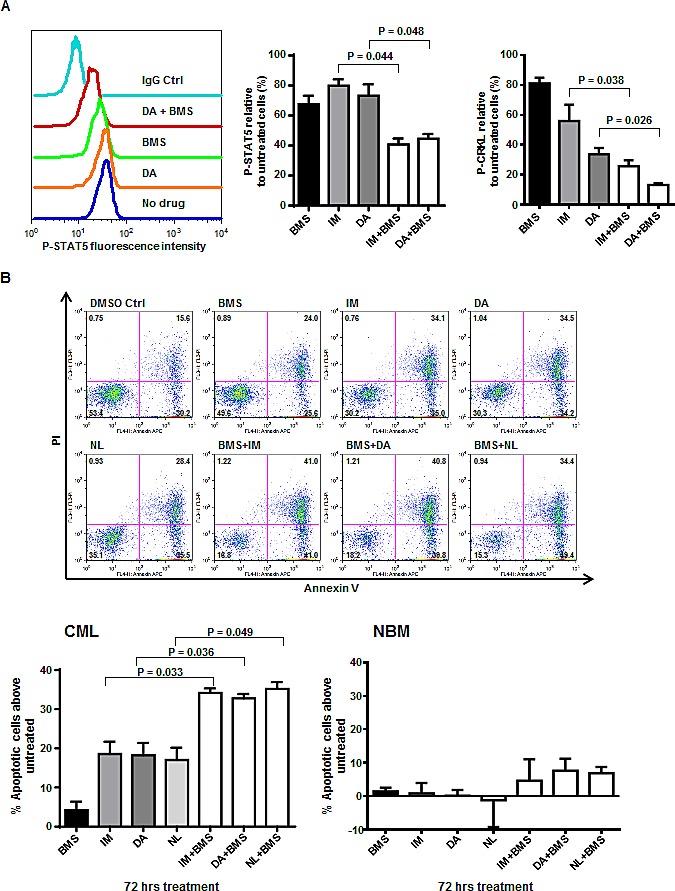
A combination of BMS-911543 and tyrosine kinase inhibitors (TKIs) results in a significant reduction in BCR-ABL and JAK2/STAT5 activities and induction of apoptosis of CD34^+^ treatment-naïve IM-nonresponder cells but not normal CD34^+^ cells (A) Phosphorylation of STAT5 and CRKL in CD34^+^ CML cells (n=4) was measured by intracellular flow cytometry after 72 hours of drug exposure, including BMS-911543 (300 nM), IM (5 μM), or dasatinib (DA, 150 nM) alone or in combination. Representative pSTAT5 fluorescence intensity histogram is shown (left panel). Phosphorylation levels were expressed as the geometric mean fluorescence intensity (MFI) subtracted by the MFI of cells stained with IgG control, and were normalized as a percentage of the untreated cells incubated with DMSO (right panel). Data shown are mean ± SEM of measurements from four individual patients. (B) Percentage of total apoptotic cells after 72 hours of drug treatments including BMS-911543 (300 nM), IM (5 μM), DA (150 nM) or nilotinib (NL, 5 μM) alone or in combination for CD34^+^ CML cells (n=3) and CD34^+^ normal bone marrow cells (NBM, n=2) as determined by Annexin V/PI staining (bottom panel). Top panel shows representative fluorescence-activated cell sorting (FACS) profiles. Data shown are mean ± SEM of measurements from three individual patients. *P* values were calculated using a two-tailed paired Student's *t* test.

To investigate whether this dual BCR-ABL-JAK2 targeting approach may also be therapeutically effective for CML patients who do not respond adequately to treatment with a single TKI, we investigated the molecular and biological effects in primitive CML cells obtained at diagnosis from CML patients (n=7) classified retrospectively following initiation of IM monotherapy, as IM-nonresponders [37, 38]. A concentration of 300 nM for BMS-911543 was selected based on the 50% inhibitory concentration (IC50) obtained in BaF3 cells transduced with a constitutively active JAK2 construct but lacking V617F mutation [35]. Notably, this concentration (300 nM) showed no toxic effects on CD34^+^ normal bone marrow (NBM) cells (~2% Annexin V positive cells, Supplementary Fig. 1A). Interestingly, intracellular staining showed that combined exposure of CD34^+^ IM-nonresponder cells (n=4) to BMS-911543 (300 nM) and a TKI (5 μM IM; 150 nM DA) produced a deeper and more prolonged suppression of p-STAT5 activity (60-65%) than IM or DA alone (20-25%) after 72 hours (P<0.05, Fig. 2A). P-CRKL activity, a direct target of BCR-ABL kinase was also suppressed more by combination treatment than single agents (70-90% vs. 45-65%, P<0.04).

While BMS-911543 (300 nM) had minimal effects on apoptosis and single TKIs (5 μM IM; 150 nM DA; 5 μM NL) increased the percentage of Annexin V positive cells by 10-20%, the combination treatment increased Annexin V positive cells to 20-30% at 48 hours, with a significant increase as a result of the combination treatment after 72 hours (2-fold, P<0.05, Fig. 2B). These results indicate that the combination of BMS-911543 and TKIs markedly and durably inhibits the activity of BCR-ABL and JAK2/STAT5 and is more effective in inducing apoptosis in IM-insensitive CML stem/progenitor cells. Critically, the combination of BMS-911543, at a clinically achievable concentration, with TKIs displayed minimal toxic effects in CD34^+^ NBM cells compared to CD34^+^ CML cells (4-8% *vs.* 32-36%, P<0.01, Fig. 2B).

To further determine whether the combination of BMS-911543 and a TKI had synergistic or addictive effect, we performed viability assays on CD34^+^ CML cells with graded doses of BMS-911543 and DA, alone or in combination, for 72 hours. The results were analyzed using CalcuSyn software (Biosoft) to calculate CI, and an algebraic estimate and a conservative isobologram were generated (Supplementary Fig. 2A and B). At doses ranging from 150 to 600 nM of BMS-911543, and from 75 to 300 nM of DA, the average CI for ED50, ED75, and ED90 was calculated to be 0.597 (Supplementary Fig. 2A), indicating that the combination is highly synergistic. The conservative isobologram analysis further confirmed synergism between the two drugs (Supplementary Fig. 2B).

### Combined exposure of a selective JAK2 inhibitor BMS-911543 and TKIs eliminates IM-insensitive CML LSCs and their progenitor cells

**Figure 3:**
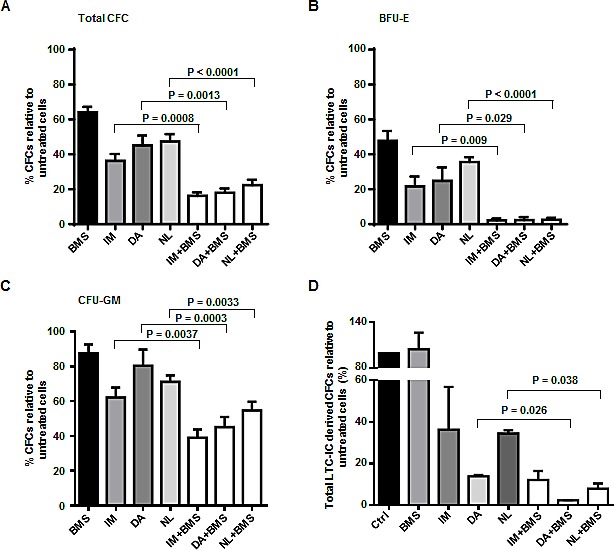
Combined exposure of BMS-911543 and TKIs eliminates CML stem and progenitor cells from IM-nonresponders (A-C) CD34^+^ CML cells (n=7) were plated in standard CFC assays plus BMS-911543 (300 nM), IM (5 μM), DA (150 nM) or NL (5 μM) alone or in combination. Colonies produced were counted after 14 days of incubation, and the numbers obtained were expressed as a percentage of values obtained in untreated cells to which only DMSO was added. The percentage of colonies containing erythroid-burst forming units (BFU-E) and granulocyte/macrophage-colony forming units (CFU-GM) are also presented (B & C). Data shown are mean ± SEM of measurements from seven individual patients. (D) CD34^+^ CML cells (n=3) were co-cultured with stromal cells and assayed for long term culture-initiating cells (LTC-ICs) in the presence of drug treatments including BMS-911543 (200 nM), IM (5 μM), DA (150 nM) or NL (5 μM) alone or in combination for two weeks. Total LTC-IC derived CFC numbers were determined from the LTCs harvested six weeks later and then expressed as a percentage of the LTC-IC CFC numbers obtained from cells in the absence of any added drug. Data shown are mean ± SEM of measurements from three individual patients. *P* values were calculated using a two-tailed paired Student's *t* test.

To further determine if combined treatment of BMS-911543 and TKIs eliminates pre-treatment LSCs and their progenitor cells from IM-nonresponders, we performed *in vitro* progenitor (CFC) and stem cell assays (LTC-IC). Combination treatment of BMS-911543 with IM, DA or NL demonstrated significant inhibition in colony growth of CD34^+^ cells compared to monotherapy alone (74-86% *vs.* 40-50% reduction, P<0.01, Fig. 3A). Notably, the combination of BMS-911543 and a TKI almost completely inhibits erythroid-burst forming unit (BFU-E) colony formation compared to TKI alone (97-98% *vs.* 65-80%, P<0.03, Fig. 3B). Granulocyte/macrophage-colony forming unit (CFU-GM) colonies were also more significantly reduced by the combination than single agents (45-60% *vs.* 20-40%, P<0.004, Fig. 3C). Furthermore, LTC-IC stem cells assays showed that the more primitive cells were more significantly eliminated by combination treatments than single agents (3-6-fold, P<0.04, Fig. 3D), indicating the potential benefit of combination therapy for targeting LSCs. Similar to the results obtained by apoptosis assay, the combination of BMS-911543 and TKIs has significantly less toxicity on CD34^+^ NBM cells than CD34^+^ CML cells (n=4, P<0.0001, Supplementary Fig. 1B). These results suggest that combination treatment with BMS-911543 and TKI is more effective in eliminating very primitive CML stem and progenitor cells from IM-nonresponders destined to develop TKI resistance.

### Combined treatment with the selective JAK2 inhibitor BMS-911543 and TKIs significantly enhances the survival of leukemic mice

**Figure 4:**
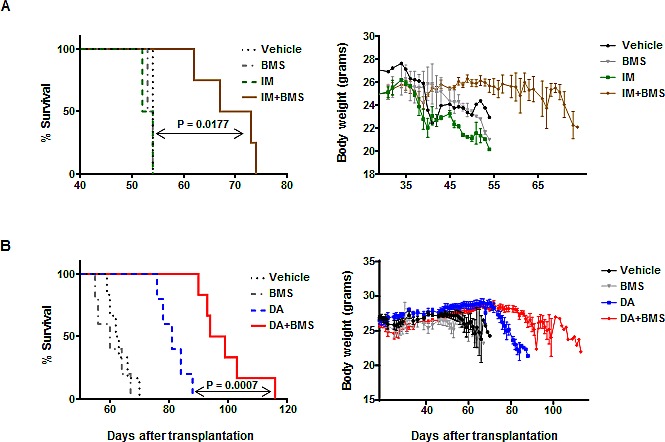
A combination of BMS-911543 and TKIs significantly enhances survival of leukemic mice CML BV173 cells (2.5×10^6^ per mouse) were intravenously injected into sub-lethally cesium-irradiated NSG mice. Two weeks after transplantation, oral gavage treatment with or without inhibitors began and continued for two weeks. (A) Survival curve for leukemic mice (n=2-4 mice per group) treated with vehicle, BMS-911543 (15 mg/kg), IM (50 mg/kg), or IM (50 mg/kg) plus BMS-911543 (15 mg/kg) twice a day for two weeks (left panel). Body weights of mice in each treatment group were measured (right panel). (B) Survival curve for leukemic mice (n=5-6 mice per group) treated with vehicle, BMS-911543 (15 mg/kg), DA (15 mg/kg), or DA (15 mg/kg) plus BMS-911543 (15 mg/kg) once a day for two weeks (left panel). Body weights of mice in each treatment group were measured (right panel). Data shown are mean ± SEM. *P* values were calculated using log-rank tests.

To assess the ability of combination treatment to eliminate primitive BCR-ABL^+^ cells with *in vivo* leukemia propagating activity, we utilized human BV173 cells, which have been shown to generate a lethal leukemia in NOD/SCID mice [22, 39]. BV173 cells (2.5x10^6^) were intravenously injected into sub-lethally irradiated NSG mice, which were then treated with inhibitors (alone or in combination) or a vehicle control by oral gavage for two weeks. In the first pilot experiment, mice were treated with vehicle control (propylene glycol), BMS-911543 (15 mg/kg), IM (50 mg/kg), and IM (50 mg/kg) plus BMS-911543 (15 mg/kg) twice a day for two weeks by oral gavage two weeks after BV173 cells injection. The combination of IM plus BMS-911543 significantly enhanced survival of leukemic mice as compared with mice treated with single agents only (median survival of IM + BMS-911543 vs. IM or BMS-911543: 70 days vs. 53 days or 54 days, P<0.02, Fig. 4A). Mice receiving combination treatment also had significantly reduced weight loss as compared with mice treated with single agents (Fig. 4A). We then investigated whether combining BMS-911543 with a more potent TKI, dasatinib (DA), might achieve better results *in vivo*. NSG mice (n = 5-6 mice per condition) injected with the same numbers of BV173 cells were treated with vehicle control, BMS-911543 (15 mg/kg), DA (15 mg/kg), and DA plus BMS-911543 once a day for two weeks by oral gavage. Significantly prolonged survival was observed in leukemic mice treated with DA plus BMS-911543 compared to DA alone (median survival of DA + BMS-911543 *vs.* DA: 96.5 days *vs.* 81 days, P=0.0007, Fig. 4B), as well as significantly reduced weight loss.

### Combination treatment with a selective JAK2 inhibitor BMS-911543 and dasatinib eradicates infiltrated leukemic cells in multiple hematopoietic tissues

**Figure 5:**
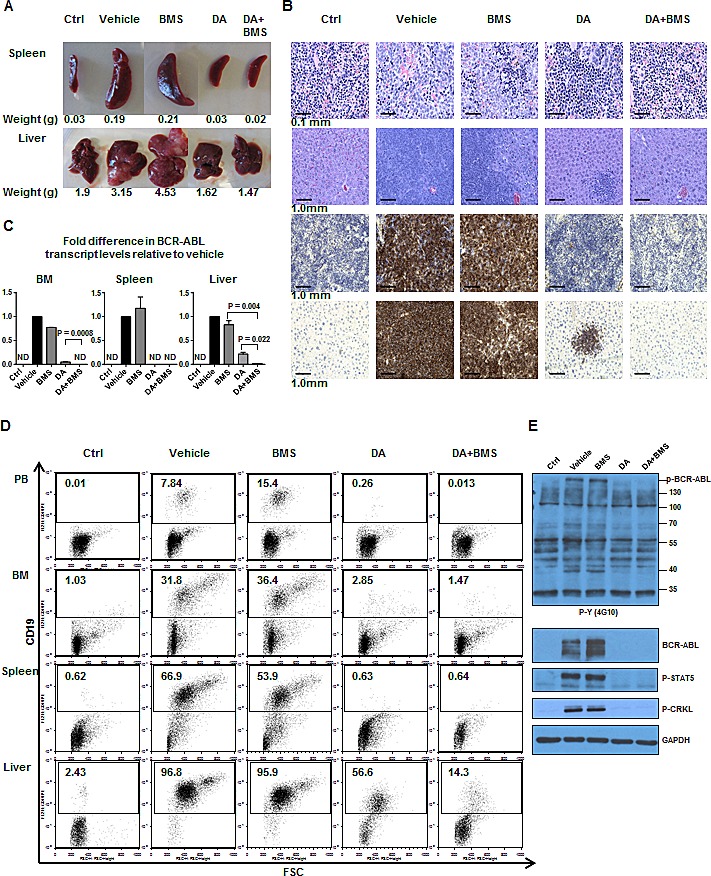
Effects of oral treatment of BMS-911543 in combination with DA on the infiltration of leukemic cells into hematopoietic tissues of mice At day 54 post-transplant, one mouse per treatment group, including no injection control (Ctrl), vehicle (no treatment), BMS-911543, DA and DA plus BMS-911543, was sacrificed and tissues were analyzed. (A) Spleen (top panel) and liver (bottom panel) weight of mice from each treatment group. (B) Hematoxylin and eosin (H&E) histology staining of spleen and liver from each treatment group (top two panels). Immunohistochemical (IHC) staining with CD19 antibody in spleen and liver (bottom two panels). (C) BCR-ABL transcript levels measured by Q-RT-PCR normalized to GAPDH. Data shown are mean ± SEM of measurements from three independent experiments. *P* values were calculated using a two-tailed paired Student's *t* test. (D) FACS profiles of engrafted human CD19^+^ cells detected in peripheral blood (PB), bone marrow (BM), spleen, and liver. (E) Western blot analysis was performed using protein lysates extracted from BM cells from each treated group and probed with specific antibodies as indicated. Ctrl = no BV173 cell injection control; ND = not detectable.

A first group of five mice transplanted with BV173 cells with or without drug treatment were sacrificed for analysis at 54 days post-transplant. We observed significantly enlarged hematopoietic organs, including spleen and liver, in mice treated with vehicle control or BMS-911543 alone but not in mice treated with DA and DA plus BMS-911543 (Fig. 5A). H&E staining revealed that mice treated with DA and DA plus BMS-911543 had no infiltration of leukemic cells to the spleen, whereas mice treated with vehicle or BMS-911543 alone had massive infiltrations of leukemic cells (Fig. 5B). DA plus BMS-911543 treated mice had no infiltration of leukemic cells to the liver, while leukemic cells were detectable in DA-treated mice. In contrast, vehicle control and BMS-911543-treated mice were heavily infiltrated with leukemic cells (Fig. 5B). IHC staining with a human CD19 antibody further confirmed that the infiltrating cells were indeed transplanted leukemic cells (Fig. 5B). Although mice treated with either DA or DA plus BMS-911543 had fewer CD19^+^ cells in PB, BM, spleen, and liver than vehicle or BMS-911543-treated mice, the effect was more pronounced in mice receiving combination treatment (Fig. 5D). Q-RT-PCR analysis further demonstrated statistically significant reduction in BCR-ABL transcript levels in mice treated with the combination compared to DA alone (BM: undetectable, P=0.0008; liver: 11-fold reduction, P=0.022, Fig. 5C). Western blot analysis demonstrated high levels of phosphorylation and protein expression of BCR-ABL, p-STAT5 and p-CRKL in BM cells from vehicle control and BMS-911543-treated mice, but undetectable levels in mice treated with DA or DA plus BMS-911543 mice (Fig. 5E).

**Figure 6:**
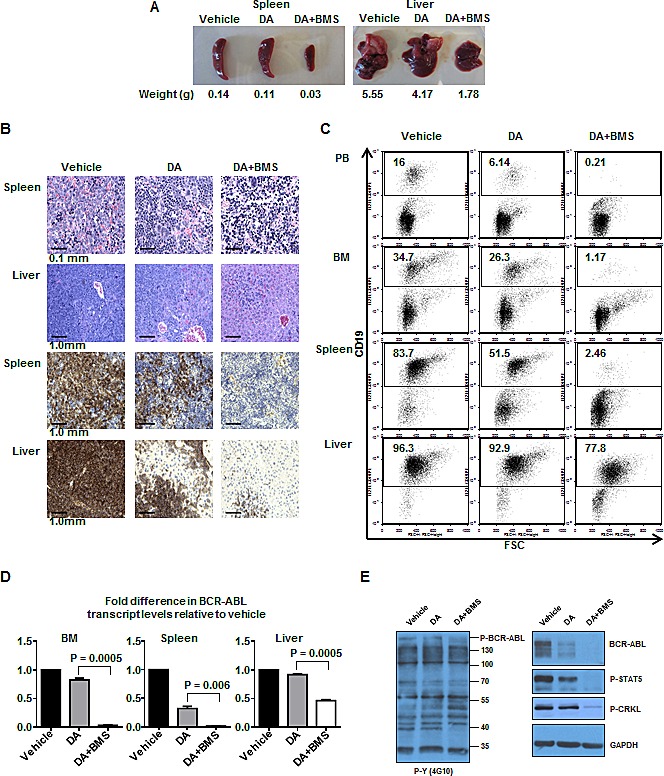
Oral treatment of BMS-911543 in combination with DA significantly eliminates infiltrated leukemic cells in hematopoietic tissues 70 days post-transplant At day 70 post-transplant, one mouse per remaining treatment group, including vehicle, DA, and DA plus BMS-911543 was sacrificed and tissues were analyzed. (A) Spleen and liver weights of mice from each treatment group. (B) H&E histology staining of spleen and liver from each treatment group (top two panels). IHC staining with CD19 antibody in spleen and liver tissues (bottom two panels). (C) FACS profiles of engrafted human CD19^+^ cells detected in PB, BM, spleen, and liver. (D) BCR-ABL transcript levels measured by Q-RT-PCR normalized to GAPDH. Data shown are mean ± SEM of measurements from three independent experiments. *P* values were calculated using a two-tailed paired Student's *t* test. (E) Western blot analysis was performed using protein lysates extracted from BM cells from each treatment group and probed with specific antibodies as indicated.

At day 70 post-transplant, the difference in infiltration of leukemic cells in hematopoietic organs between mice injected with DA alone or DA plus BMS-911543 was more pronounced. Enlarged spleens and livers were observed in mice treated with DA alone, but not in mice treated with DA plus BMS-911543 (Fig. 6A). H&E and IHC staining of spleen and liver revealed increased infiltration of leukemic cells in DA or vehicle-treated mice, but very low levels in DA plus BMS-911543-treated mice (Fig. 6B). In addition, mice receiving combination treatment showed significantly reduced engraftment levels in PB, BM, and spleen compared to mice treated with DA alone (0.2% *vs.* 6.1%, 1.2% *vs.* 26%, and 2.5% vs. 52%, Fig. 6C). It was observed that relatively higher levels of engrafted cells occurred in the liver as compared to other tissues under the combination treatment. Finally, BCR-ABL transcript levels in mice treated with DA plus BMS-911543 were much lower than mice treated with DA alone (BM: 20-fold reduction, P=0.0005; spleen: 32-fold reduction, P=0.006; liver: 2-fold reduction, P=0.0005; Fig. 6D). Western blot analysis further showed highly increased levels of phosphorylation and protein expression of BCR-ABL, p-STAT5 and p-CRKL in mice treated with DA alone compared to DA plus BMS-911543 (Fig. 6E). Taken together, these results suggest that the oral combination treatment is much more effective than either agent alone in eliminating primitive human CML cells able to generate aggressive leukemia in mice, with significantly enhanced survival of leukemic mice.

## DISCUSSION

In this study, we provide pre-clinical evidence that combination treatment with a selective JAK2 inhibitor (BMS-911543) and TKI more effectively eradicates IM-insensitive BCR-ABL^+^ cells and primary CML stem/progenitor cells compared to either BMS-911543 or TKI alone, suggesting a potential new treatment option for patients with CML. Specifically, we examined whether combination treatment might be a much better strategy for CML patients who are unlikely to respond to TKI monotherapies. These patients might benefit from such a treatment, which could more effectively reduce the CML stem cell burden, avoiding the development of TKI-resistance and disease relapse. Our study on CD34^+^ treatment-naive IM-nonresponder cells supports this hypothesis. We demonstrated that BMS-911543, at clinically achievable concentrations, in combination with a TKI markedly reduced the output of progenitor colonies and eradicated their more primitive stem cells *in vitro* (Fig. 3). The combination was also more effective at reducing p-CRKL and p-STAT5 activities in these cells at the molecular level (Fig. 2). In addition, the combination treatment displays synergism, suggesting that simultaneously targeting BCR-ABL and JAK2 activities in CML stem/progenitor cells is indeed more effective than using single agents.

Since transplantation of primary CML stem/progenitor cells is not able to generate leukemia in immunodeficient mice [22], we screened several BCR-ABL^+^ human cell lines to determine which could generate leukemia *in vivo* and found that human BV173 cells, but not K562 cells, are capable of infiltrating into multiple hematopoietic organs and generating a lethal leukemia in NSG mice. Thus, this is a useful model for examining efficacy of drug treatment in BCR-ABL^+^ human cells *in vivo.* Indeed, *in vivo* oral administration of BMS-911543 and TKI for two weeks significantly eliminated infiltrated leukemic cells to a greater extent in multiple hematopoietic tissues than TKI monotherapy (Fig. 4-6). A statistically significant prolonged survival of treated mice was obtained using the combination treatment, whereas IM or BMS-911543 alone was ineffective at preventing disease development. Compared with IM, dasatinib is not only a dual SRC-ABL inhibitor, but it is also 300-fold more potent in inhibiting ABL kinase *in vitro*, and induces much greater and faster rates of major molecular response in patients. [7, 40, 41]. Therefore, treatment with the potent TKI dasatinib alone appears to be more effective at prolonging disease survival than IM alone, and the combination of BMS-911543 and dasatinib even more significantly enhances survival of leukemic mice and prevents infiltration of leukemic cells in multiple hematopoietic tissues. Our study suggests a new strategy for treating CP-CML patients at risk of developing TKI resistance and for targeting more aggressive leukemic cells present in later stage CML patients, which are routinely only poorly responsive to TKI monotherapy [10, 11].

Consistent with our studies, it has been reported that disrupting the BCR-ABL/JAK2-STAT5 network eliminates BCR-ABL-transduced cells and primitive CD34^+^ CML cells; JAK2 inhibitors have been shown to sensitize CML cells to TKIs in the BM microenvironment [42-44]. Knockdown of JAK2 using a shRNA approach reduced BCR-ABL expression, which further downregulated STAT5 activity [20], and suppression of JAK2 also reduced protein levels of β-catenin protein [45], possibly through activation of GSK-3β [21]. In addition, JAK2 siRNA knockdown reduced BCR-ABL-mediated c-Myc expression [21, 46]. On the other hand, it was reported that BCR-ABL directly phosphorylates STAT5 in BCR-ABL-transduced cells [25], and that Jak2 is not required for initial myeloid transformation and leukemia maintenance in a Jak2 conditional knockout model [47]. This discrepancy could be due to the different model systems and cell types used in these studies. For example, it is known that BCR-ABL*-*transduced murine BM cells, transduced using a retroviral transduction model, express much higher levels of BCR-ABL than physiologically primary CML cells from patients’ blood or BM samples and that the role of JAK2 in enhancing cell survival might not be required in these BCR-ABL over-expressing cells, rendering JAK2 dispensable. Indeed, increasing evidence indicates that the canonical JAK2/STAT5 pathway is critical for primary CML stem/progenitor cells, which rely on cytokine-activated JAK2/STAT5 signaling in addition to BCR-ABL signaling [34, 43, 44, 48, 49]. It has been reported that activation of BCR-ABL stimulates the production of cytokines, including IL-3, G-CSF and GM-CSF, which bind to their cognate receptors and contribute to TKI resistance of CML stem/progenitor cells through activation of the JAK2/STAT5 pathway [44, 48]. It was also reported that BCR-ABL interacts with the IL-3/GM-CSF receptor, which leads to the downstream activation of JAK2 [49], and that blockage of JAK2-mediated extrinsic survival signals using JAK2 inhibitors restores sensitivity of CML cells to TKIs [43]. We and others have shown that destabilization of the BCR-ABL/JAK2 network with JAK2 inhibitors and TKIs dissociates their physical interaction and sensitizes CML LSCs to TKIs. These effects are not observed in the same cells treated with TKIs alone or even with combination of TKIs [20-22, 42]. Consequently, these findings provide compelling evidence that JAK2/STAT5 signaling represents an important node supporting CML LSC growth and survival. Targeting BCR-ABL-JAK2 cooperative activities may reverse the innate TKI-resistance phenotype of CML LSCs and sensitize them to TKI.

BMS-911543 is a highly-selective JAK2 inhibitor which has an IC_50_ of 1.1 nM with little effects on JAK1 (356 nM), JAK3 (73 nM), or TYK2 (66 nM), respectively [35]. It is highly specific; no other non-JAK family targets with an IC_50_ of less than 100 nM have been reported [29]. It is currently being investigated in a Phase 1/2a clinical trial in myelofibrosis (ClinicalTrals.gov identifier: NCT01236352) [33]. It thus provides a rational basis for a therapeutic combination strategy applying BMS-911543 and a TKI in CML. We found that BMS-911543 alone had limited inhibitory effects on primary CML stem/progenitor cells when its concentration was non-toxic to primitive healthy BM cells. Similarly, no significant changes in phosphorylation levels of the BCR-ABL kinase substrate CRKL were observed when these cells were treated with BMS-911543 monotherapy. This suggests specific inhibition of JAK2 downstream of BCR-ABL, and its effects can be significantly enhanced by combining with a TKI. This observation also reduces concerns of off-target effects of BMS-911543 on other kinases and signaling proteins that result in toxicity and have been reported for several other JAK2 inhibitors [29, 33, 47, 50, 51]. Taken together, this pre-clinical study provides strong scientific rational for the continued investigation of JAK2 inhibition as a therapeutic strategy in CML and demonstrates the potential merit of combining a highly JAK2-specific inhibitor with potent TKIs to specifically target CML stem/progenitor cells, especially in CML patients likely to develop TKI-resistance if treated with TKI monotherapy.

## METHODS

### Human cells

Heparin-anticoagulated peripheral blood (PB) cells were obtained from newly-diagnosed patients prior to TKI therapy that were clinically classified, following IM monotherapy, as IM-nonresponders, based on the European Leukemia Net treatment guidelines [37, 38]. Bone marrow (NBM) cells were also obtained from healthy adult donors. Informed consent was obtained in accordance with the Declaration of Helsinki, and the procedures used approved by the Research Ethics Board at the University of British Columbia. Mononuclear cells were isolated using Ficoll-Hypaque (Sigma-Aldrich) density gradient separation and CD34^+^ cells (>85%) were enriched immunomagnetically using the EasySep CD34 positive selection kit (STEMCELL Technologies). Purity was verified using fluorescence-activated cell sorter (FACS) analysis as described [22].

### Reagents

BMS-911543 and dasatinib (DA) were provided by Bristol-Myers Squibb (Princeton, USA). IM and nilotinib (NL) were obtained from Novartis (Novartis, Basel, Switzerland). Stock solutions of 10 mM were prepared with water (IM) or with dimethyl sulfoxide (DMSO) (DA, NL, and BMS-911543) and stored at -20°C.

### Suspension cultures and analysis of drug interactions

CD34^+^ cells were cultured in Iscove's medium plus bovine serum albumin (BSA), insulin, transferrin (STEMCELL Technologies) and 10^-4^ M 2-mercaptoethanol supplemented with four growth factors (20 ng/mL IL-3, 20 ng/mL IL-6, 100ng/mL Flt3-ligand, and 20 ng/mL G-CSF), with or without drug treatments. CML cell lines were maintained in RPMI 1640 media as described [22]. Cell counts and viability were assessed using trypan blue dye exclusion. Analysis of drug interactions in the combination treatment group of BMS-911543 with DA was assessed after 72 hours of suspension culture with drug exposure using total viable cell counts. Data were analyzed using constant-ratio drug combinations and the median-effect method of Chou and Talalay [52]. The combination Index (CI) was calculated using CalcuSyn software (Biosoft, Cambridge, United Kingdom). CI< 1, CI = 1 or CI > 1 represent synergistic, additive or antagonistic effects respectively.

### Intracellular staining

After 72 hours of suspension culture with drug exposure, CD34^+^ CML cells were stained with p-CRKL (Cell Signaling) or p-STAT5 antibodies (Cell Signaling) overnight, followed by incubation with a secondary antibody (anti–rabbit IgG FITC-conjugate, Invitrogen) prior to FACS analysis as previously described [53]. P-CRKL and p-STAT5 levels were determined as the geometric mean fluorescence intensity (MFI) subtracted by the MFI of cells stained with isotype IgG control, and were normalized as a percentage of control cells incubated for the same time with DMSO only.

### Apoptosis assays

After 48 and 72 hours of suspension culture with drug treatments, apoptosis assays were performed on CD34^+^ NBM cells and CD34^+^ CML cells using an Annexin V Apoptosis Detection Kit APC (eBioscience). Briefly, cells were stained with Annexin V APC and Propidium Iodide (PI) and analyzed using a FACSCalibur (BD Bioscience). Total apoptotic cell populations were determined as the sum of the “early” apoptotic cells (Annexin V^+^ only) and “late” apoptotic cells (Annexin V^+^/PI^+^).

### Colony-forming cell (CFC) and long-term culture-initiating cell (LTC-IC) assays

CFC assays were performed as previously described [54]. Briefly, 600 K562R cells, 1,500 BV173 or 3,000 CD34^+^ cells were mixed in 3 mL methylcellulose medium (STEMCELL Technologies) with or without inhibitors. Colonies produced were counted after 14 to 16 days of incubation. LTC-IC assays were performed with CD34^+^ cells on M2-10B4 stromal cells as previously described [22]. Cultures were maintained for six weeks with weekly half-medium changes, plus inhibitors for two weeks. Cells were then harvested and CFC assays performed to obtain total numbers of LTC-IC-derived CFCs.

### Transplantation of immunodeficient mice with CML cells

BV173 cells (2.5 × 10^6^ cells per treatment condition) were injected intravenously into 8- to 10-week-old, sub-lethally cesium irradiated (315 cGy) NOD/SCID-interleukin 2 receptor γ–chain-deficient (NSG) mice. Two weeks post-transplant, mice were treated with vehicle and inhibitors once or twice a day for two weeks by oral gavage. Mice were monitored daily for body weight changes and survival during and after treatment. The level of engraftment of BV173 cells in the PB, BM, spleen, and liver was determined with anti-human CD19PE antibody (1:250 dilution) (eBioscience) and FACS analysis. For histopathology analysis, spleens and livers were fixed in 10% neutral buffered formalin, embedded in paraffin, and stained with hematoxylin and eosin (H-&-E), or with anti-human CD19 antibody (Abcam) for immunohistochemical (IHC) staining. Images of histological slides were captured on a Zeiss Axioplan 2 Imaging microscope (Göttingen, Germany) equipped with a Retiga EXi colour digital camera (Burnaby, Canada). Animal experiments were performed in the Animal Resource Centre of the BC Cancer Agency Research Centre, using procedures approved by the Animal Care Committee of the University of British Columbia (Vancouver).

### RNA extraction and quantitative real-time PCR

Total RNA was extracted with TRIzol (Life Technologies) [55]. Glycogen (10μg/ml, Life Technologies) was added as a carrier to facilitate visibility of the RNA pellet. RNA (100 ng) was reversed transcribed into cDNA with SuperScript® VILO^TM^ Master Mix (Life Technologies). Quantitative real-time PCR was performed and specific primers to detect BCR-ABL transcripts were as previously described [15].

### Western blotting analysis

Cells were lysed in protein solubilization buffer (PSB) and analyzed by Western blotting as previously described [54]. Antibodies used were anti-human STAT5 (Millipore), anti-phospho-STAT5 (Cell Signaling), anti-phospho-tyrosine (4G10, Millipore), anti-ABL (8E9, BD Biosciences), anti-phospho-CRKL (Cell Signaling), and anti-human GAPDH (Sigma Aldrich).

### Statistical analysis

Results are shown as the mean ± standard error of the mean (SEM) of measurements from at least three independent experiments. Differences between groups were compared using the two-tailed Student's t test for paired samples, or one-way ANOVA with post-hoc testing for multiple comparisons. Log-rank tests were used to compare the median survival of mice from different groups. All statistical analyses were performed using GraphPad Prism version 6 (http://www.graphpad.com/scientific-software/prism/). P-values <0.05 were considered statistically significant.

## SUPPLEMENTARY MATERIAL AND FIGURES



## References

[1] Goldman JM, Melo JV (2003). Chronic myeloid leukemia--advances in biology and new approaches to treatment. The New England journal of medicine.

[2] Sloma I, Jiang X, Eaves AC, Eaves CJ (2010). Insights into the stem cells of chronic myeloid leukemia. Leukemia.

[3] Sattler M, Griffin JD (2003). Molecular mechanisms of transformation by the BCR-ABL oncogene. Seminars in hematology.

[4] Van Etten RA (2007). Oncogenic signaling: new insights and controversies from chronic myeloid leukemia. The Journal of experimental medicine.

[5] Druker BJ, Guilhot F, O'Brien SG, Gathmann I, Kantarjian H, Gattermann N, Deininger MW, Silver RT, Goldman JM, Stone RM, Cervantes F, Hochhaus A, Powell BL, Gabrilove JL, Rousselot P, Reiffers J (2006). Five-year follow-up of patients receiving imatinib for chronic myeloid leukemia. The New England journal of medicine.

[6] Hughes TP, Lipton JH, Spector N, Cervantes F, Pasquini R, Clementino NC, Dorlhiac Llacer PE, Schwarer AP, Mahon FX, Rea D, Branford S, Purkayastha D, Collins L, Szczudlo T, Leber B (2014). Deep molecular responses achieved in patients with CML-CP who are switched to nilotinib after long-term imatinib. Blood.

[7] Shah NP, Tran C, Lee FY, Chen P, Norris D, Sawyers CL (2004). Overriding imatinib resistance with a novel ABL kinase inhibitor. Science.

[8] Kantarjian H, le Coutre P, Cortes J, Pinilla-Ibarz J, Nagler A, Hochhaus A, Kimura S, Ottmann O (2010). Phase 1 study of INNO-406, a dual Abl/Lyn kinase inhibitor, in Philadelphia chromosome-positive leukemias after imatinib resistance or intolerance. Cancer.

[9] Weisberg E, Manley PW, Breitenstein W, Bruggen J, Cowan-Jacob SW, Ray A, Huntly B, Fabbro D, Fendrich G, Hall-Meyers E, Kung AL, Mestan J, Daley GQ, Callahan L, Catley L, Cavazza C (2005). Characterization of AMN107, a selective inhibitor of native and mutant Bcr-Abl. Cancer cell.

[10] Gorre ME, Mohammed M, Ellwood K, Hsu N, Paquette R, Rao PN, Sawyers CL (2001). Clinical resistance to STI-571 cancer therapy caused by BCR-ABL gene mutation or amplification. Science.

[11] O'Hare T, Zabriskie MS, Eiring AM, Deininger MW (2012). Pushing the limits of targeted therapy in chronic myeloid leukaemia. Nature reviews Cancer.

[12] Mahon FX, Rea D, Guilhot J, Guilhot F, Huguet F, Nicolini F, Legros L, Charbonnier A, Guerci A, Varet B, Etienne G, Reiffers J, Rousselot P (2010). Discontinuation of imatinib in patients with chronic myeloid leukaemia who have maintained complete molecular remission for at least 2 years: the prospective, multicentre Stop Imatinib (STIM) trial. The lancet oncology.

[13] Rousselot P, Charbonnier A, Cony-Makhoul P, Agape P, Nicolini FE, Varet B, Gardembas M, Etienne G, Rea D, Roy L, Escoffre-Barbe M, Guerci-Bresler A, Tulliez M, Prost S, Spentchian M, Cayuela JM (2014). Loss of major molecular response as a trigger for restarting tyrosine kinase inhibitor therapy in patients with chronic-phase chronic myelogenous leukemia who have stopped imatinib after durable undetectable disease. Journal of clinical oncology : official journal of the American Society of Clinical Oncology.

[14] Chu S, McDonald T, Lin A, Chakraborty S, Huang Q, Snyder DS, Bhatia R (2011). Persistence of leukemia stem cells in chronic myelogenous leukemia patients in prolonged remission with imatinib treatment. Blood.

[15] Jiang X, Saw KM, Eaves A, Eaves C (2007). Instability of BCR-ABL gene in primary and cultured chronic myeloid leukemia stem cells. Journal of the National Cancer Institute.

[16] Jiang X, Zhao Y, Smith C, Gasparetto M, Turhan A, Eaves A, Eaves C (2007). Chronic myeloid leukemia stem cells possess multiple unique features of resistance to BCR-ABL targeted therapies. Leukemia.

[17] Zhang H, Li S (2013). Molecular mechanisms for survival regulation of chronic myeloid leukemia stem cells. Protein & cell.

[18] Corbin AS, Agarwal A, Loriaux M, Cortes J, Deininger MW, Druker BJ (2011). Human chronic myeloid leukemia stem cells are insensitive to imatinib despite inhibition of BCR-ABL activity. The Journal of clinical investigation.

[19] Hamilton A, Helgason GV, Schemionek M, Zhang B, Myssina S, Allan EK, Nicolini FE, Muller-Tidow C, Bhatia R, Brunton VG, Koschmieder S, Holyoake TL (2012). Chronic myeloid leukemia stem cells are not dependent on Bcr-Abl kinase activity for their survival. Blood.

[20] Samanta A, Perazzona B, Chakraborty S, Sun X, Modi H, Bhatia R, Priebe W, Arlinghaus R (2011). Janus kinase 2 regulates Bcr-Abl signaling in chronic myeloid leukemia. Leukemia.

[21] Samanta AK, Lin H, Sun T, Kantarjian H, Arlinghaus RB (2006). Janus kinase 2: a critical target in chronic myelogenous leukemia. Cancer research.

[22] Chen M, Gallipoli P, DeGeer D, Sloma I, Forrest DL, Chan M, Lai D, Jorgensen H, Ringrose A, Wang HM, Lambie K, Nakamoto H, Saw KM, Turhan A, Arlinghaus R, Paul J (2013). Targeting primitive chronic myeloid leukemia cells by effective inhibition of a new AHI-1-BCR-ABL-JAK2 complex. Journal of the National Cancer Institute.

[23] Esmailzadeh S, Jiang X (2011). AHI-1: a novel signaling protein and potential therapeutic target in human leukemia and brain disorders. Oncotarget.

[24] Carlesso N, Frank DA, Griffin JD (1996). Tyrosyl phosphorylation and DNA binding activity of signal transducers and activators of transcription (STAT) proteins in hematopoietic cell lines transformed by Bcr/Abl. The Journal of experimental medicine.

[25] Ilaria RL (1996). and Van Etten RA. P210 and P190(BCR/ABL) induce the tyrosine phosphorylation and DNA binding activity of multiple specific STAT family members. The Journal of biological chemistry.

[26] Moriggl R, Sexl V, Kenner L, Duntsch C, Stangl K, Gingras S, Hoffmeyer A, Bauer A, Piekorz R, Wang D, Bunting KD, Wagner EF, Sonneck K, Valent P, Ihle JN, Beug H (2005). Stat5 tetramer formation is associated with leukemogenesis. Cancer cell.

[27] Nelson EA, Walker SR, Weisberg E, Bar-Natan M, Barrett R, Gashin LB, Terrell S, Klitgaard JL, Santo L, Addorio MR, Ebert BL, Griffin JD, Frank DA (2011). The STAT5 inhibitor pimozide decreases survival of chronic myelogenous leukemia cells resistant to kinase inhibitors. Blood.

[28] Warsch W, Kollmann K, Eckelhart E, Fajmann S, Cerny-Reiterer S, Holbl A, Gleixner KV, Dworzak M, Mayerhofer M, Hoermann G, Herrmann H, Sillaber C, Egger G, Valent P, Moriggl R, Sexl V (2011). High STAT5 levels mediate imatinib resistance and indicate disease progression in chronic myeloid leukemia. Blood.

[29] Tefferi A (2012). JAK inhibitors for myeloproliferative neoplasms: clarifying facts from myths. Blood.

[30] Pardanani A, Gotlib JR, Jamieson C, Cortes JE, Talpaz M, Stone RM, Silverman MH, Gilliland DG, Shorr J, Tefferi A (2011). Safety and efficacy of TG101348, a selective JAK2 inhibitor, in myelofibrosis. Journal of clinical oncology : official journal of the American Society of Clinical Oncology.

[31] Wernig G, Kharas MG, Okabe R, Moore SA, Leeman DS, Cullen DE, Gozo M, McDowell EP, Levine RL, Doukas J, Mak CC, Noronha G, Martin M, Ko YD, Lee BH, Soll RM (2008). Efficacy of TG101348, a selective JAK2 inhibitor, in treatment of a murine model of JAK2V617F-induced polycythemia vera. Cancer cell.

[32] Cook AM, Li L, Ho Y, Lin A, Stein A, Forman S, Perrotti D, Jove R, Bhatia R (2014). Role of altered growth factor receptor mediated JAK2 signaling in growth and maintenance of human acute myeloid leukemia stem cells. Blood.

[33] Tam CS, Verstovsek S (2013). Investigational Janus kinase inhibitors. Expert opinion on investigational drugs.

[34] Warsch W, Walz C, Sexl V (2013). JAK of all trades: JAK2-STAT5 as novel therapeutic targets in BCR-ABL1+ chronic myeloid leukemia. Blood.

[35] Purandare AV, McDevitt TM, Wan H, You D, Penhallow B, Han X, Vuppugalla R, Zhang Y, Ruepp SU, Trainor GL, Lombardo L, Pedicord D, Gottardis MM, Ross-Macdonald P, de Silva H, Hosbach J (2012). Characterization of BMS-911543, a functionally selective small-molecule inhibitor of JAK2. Leukemia.

[36] Deutsch E, Maggiorella L, Wen B, Bonnet ML, Khanfir K, Frascogna V, Turhan AG, Bourhis J (2004). Tyrosine kinase inhibitor AG1024 exerts antileukaemic effects on STI571-resistant Bcr-Abl expressing cells and decreases AKT phosphorylation. British journal of cancer.

[37] Forrest DL, Jiang X, Eaves CJ, Smith CL (2008). An approach to the management of chronic myeloid leukemia in British Columbia. Curr Oncol.

[38] Baccarani M, Cortes J, Pane F, Niederwieser D, Saglio G, Apperley J, Cervantes F, Deininger M, Gratwohl A, Guilhot F, Hochhaus A, Horowitz M, Hughes T, Kantarjian H, Larson R, Radich J (2009). Chronic myeloid leukemia: an update of concepts and management recommendations of European LeukemiaNet. J Clin Oncol.

[39] Dazzi F, Capelli D, Hasserjian R, Cotter F, Corbo M, Poletti A, Chinswangwatanakul W, Goldman JM, Gordon MY (1998). The kinetics and extent of engraftment of chronic myelogenous leukemia cells in non-obese diabetic/severe combined immunodeficiency mice reflect the phase of the donor's disease: an *in vivo* model of chronic myelogenous leukemia biology. Blood.

[40] Kantarjian H, Shah NP, Hochhaus A, Cortes J, Shah S, Ayala M, Moiraghi B, Shen Z, Mayer J, Pasquini R, Nakamae H, Huguet F, Boque C, Chuah C, Bleickardt E, Bradley-Garelik MB (2010). Dasatinib versus imatinib in newly diagnosed chronic-phase chronic myeloid leukemia. The New England journal of medicine.

[41] Lombardo LJ, Lee FY, Chen P, Norris D, Barrish JC, Behnia K, Castaneda S, Cornelius LA, Das J, Doweyko AM, Fairchild C, Hunt JT, Inigo I, Johnston K, Kamath A, Kan D (2004). Discovery of N-(2-chloro-6-methyl- phenyl)-2-(6-(4-(2-hydroxyethyl)- piperazin-1-yl)-2-methylpyrimidin-4- ylamino)thiazole-5-carboxamide (BMS-354825), a dual Src/Abl kinase inhibitor with potent antitumor activity in preclinical assays. Journal of medicinal chemistry.

[42] Samanta AK, Chakraborty SN, Wang Y, Kantarjian H, Sun X, Hood J, Perrotti D, Arlinghaus RB (2009). Jak2 inhibition deactivates Lyn kinase through the SET-PP2A-SHP1 pathway, causing apoptosis in drug-resistant cells from chronic myelogenous leukemia patients. Oncogene.

[43] Traer E, MacKenzie R, Snead J, Agarwal A, Eiring AM, O'Hare T, Druker BJ, Deininger MW (2012). Blockade of JAK2-mediated extrinsic survival signals restores sensitivity of CML cells to ABL inhibitors. Leukemia.

[44] Wang Y, Cai D, Brendel C, Barett C, Erben P, Manley PW, Hochhaus A, Neubauer A, Burchert A (2007). Adaptive secretion of granulocyte-macrophage colony-stimulating factor (GM-CSF) mediates imatinib and nilotinib resistance in BCR/ABL+ progenitors via JAK-2/STAT-5 pathway activation. Blood.

[45] Neviani P, Harb JG, Oaks JJ, Santhanam R, Walker CJ, Ellis JJ, Ferenchak G, Dorrance AM, Paisie CA, Eiring AM, Ma Y, Mao HC, Zhang B, Wunderlich M, May PC, Sun C (2013). PP2A-activating drugs selectively eradicate TKI-resistant chronic myeloid leukemic stem cells. The Journal of clinical investigation.

[46] Xie S, Lin H, Sun T, Arlinghaus RB (2002). Jak2 is involved in c-Myc induction by Bcr-Abl. Oncogene.

[47] Hantschel O, Warsch W, Eckelhart E, Kaupe I, Grebien F, Wagner KU, Superti-Furga G, Sexl V (2012). BCR-ABL uncouples canonical JAK2-STAT5 signaling in chronic myeloid leukemia. Nature chemical biology.

[48] Jiang X, Lopez A, Holyoake T, Eaves A, Eaves C (1999). Autocrine production and action of IL-3 and granulocyte colony-stimulating factor in chronic myeloid leukemia. Proceedings of the National Academy of Sciences of the United States of America.

[49] Wilson-Rawls J, Xie S, Liu J, Laneuville P, Arlinghaus RB (1996). P210 Bcr-Abl interacts with the interleukin 3 receptor beta(c) subunit and constitutively induces its tyrosine phosphorylation. Cancer research.

[50] Ratner M (2014). Setback for JAK2 inhibitors. Nature biotechnology.

[51] Zhou T, Georgeon S, Moser R, Moore DJ, Caflisch A, Hantschel O (2014). Specificity and mechanism-of-action of the JAK2 tyrosine kinase inhibitors ruxolitinib and SAR302503 (TG101348). Leukemia.

[52] Chou TC, Talalay P (1984). Quantitative analysis of dose-effect relationships: the combined effects of multiple drugs or enzyme inhibitors. Advances in enzyme regulation.

[53] Hamilton A, Elrick L, Myssina S, Copland M, Jorgensen H, Melo JV, Holyoake T (2006). BCR-ABL activity and its response to drugs can be determined in CD34+ CML stem cells by CrkL phosphorylation status using flow cytometry. Leukemia.

[54] Zhou LL, Zhao Y, Ringrose A, DeGeer D, Kennah E, Lin AE, Sheng G, Li XJ, Turhan A, Jiang X (2008). AHI-1 interacts with BCR-ABL and modulates BCR-ABL transforming activity and imatinib response of CML stem/progenitor cells. The Journal of experimental medicine.

[55] Rio DC, Ares M, Hannon GJ, Nilsen TW (2010). Purification of RNA using TRIzol (TRI reagent). Cold Spring Harb Protoc.

