# Supplementary material for: Selective JAK2/ABL dual inhibition therapy effectively eliminates TKI-insensitive CML stem/progenitor cells
Source: Oncotarget. 2014 Aug 16;5(18):8637–50. doi: 10.18632/oncotarget.2353 (PMC4226710; doi:10.18632/oncotarget.2353)
Supplement: Supplementary file 1 [file oncotarget-05-8637-s001.pdf]

Selective JAK2/ABL dual inhibition therapy effectively eliminates TKI-insensitive CML stem/progenitor cells

Supplementary Material

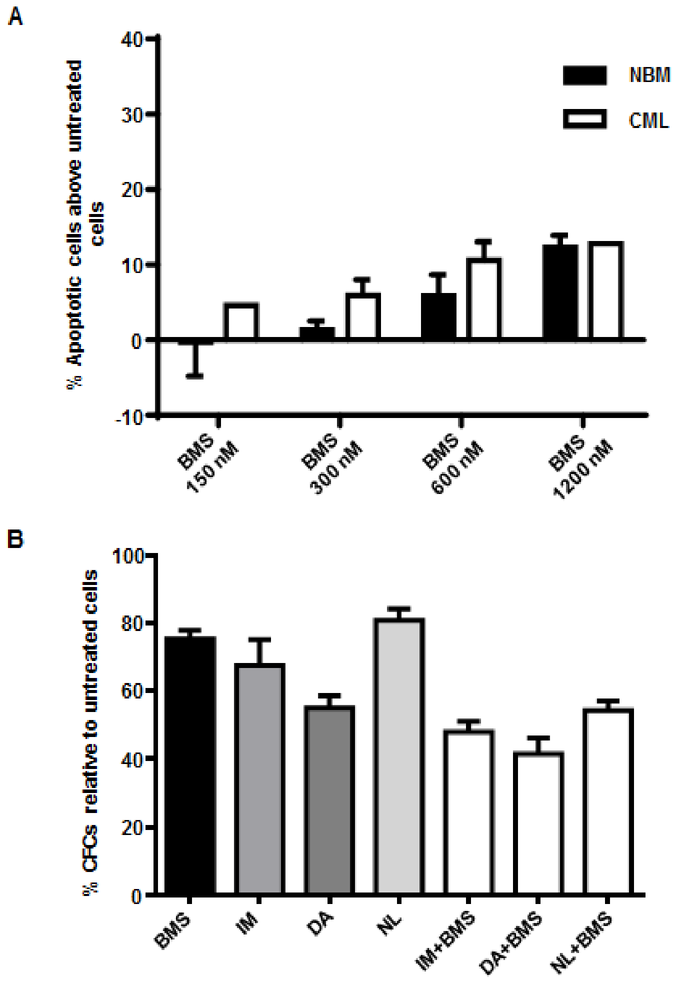

Figure S1: Effects of BMS-911543 alone or in combination with TKIs on normal CD34$^+$ bone marrow cells. (A) Percentage of total apoptotic cells determined by Annexin V/PI staining after 72 hours of BMS-911543 treatments (150 nM, 300 nM, 600 nM, or 1200 nM) in CD34$^+$ normal bone marrow cells (NBM, n=2) and CD34$^+$ CML cells (n=2). (B) CD34$^+$ NBM cells (n=4) were plated in standard CFC assays plus BMS (300 nM), IM (5 µM), DA (150 nM) or NL (5 µM) alone or in combination. Colonies produced were counted after 14 days of incubation, and the numbers obtained were expressed as a percentage of values obtained in untreated cells to which only DMSO was added.

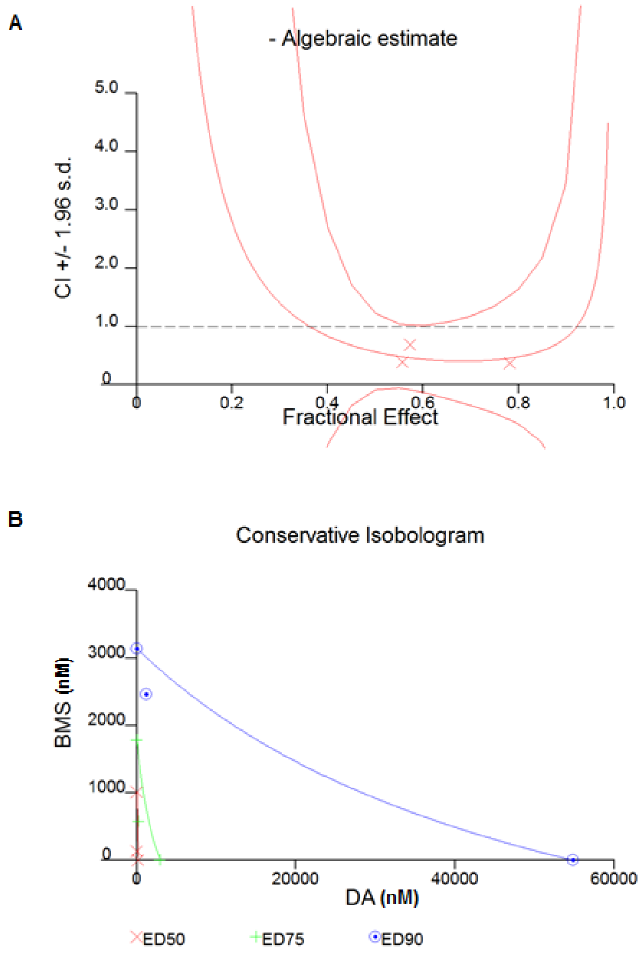

Figure S2: Efficacy of drug interactions by combined treatment with BMS-911543 and DA against CD34$^+$ CML cells. (A) Drug interactions for the combination of BMS-911543 with DA were assessed with drug exposure (BMS: 150 nM, 300 nM, 600 nM; DA: 75 nM, 150 nM, 300 nM; or DA + BMS) against CD34$^+$ CML cells by viability assays after 72 hours of drug exposure. CI plots for BMS and DA were calculated using CalcuSyn software in an algebraic estimate. The combination of BMS plus DA showed synergistic activity (CI values <1) in CD34$^+$ CML cells. (B) A conservative isobologram analysis for CD34$^+$ CML cells indicated synergism between BMS and DA.
